# Gaps in Knowledge and Practice of Cervical Cancer Screening among Primary Health Care Workers in Lagos State, Nigeria: A Cross-Sectional Study

**DOI:** 10.12688/f1000research.180107.1

**Published:** 2026-04-28

**Authors:** Adebola Afolake Adejimi, Olayemi Dawodu, Adaiah P Soibi-Harry, Adewumi Alabi, Ololade Onasanya, Blossom Adaeze Maduafokwa, Kamaldeen Sunkanmi Abdulraheem, Ibraheem Shola Abdulraheem, Oluwafisayo Oluwaseun Omotayo, Kehinde Sharafadeen Okunade

**Affiliations:** 1 Department of Community Health and Primary Care, University of Lagos College of Medicine, Lagos, Lagos State, Nigeria; 2 Department of Community Health and Primary Care, Lagos University Teaching Hospital, Surulere, Lagos, Lagos State, Nigeria; 3 Department of Anatomic and Molecular Pathology, University of Lagos College of Medicine, Lagos, Lagos State, Nigeria; 4 Department of Obstetrics and Gynecology, Lagos University Teaching Hospital, Surulere, Lagos, Lagos State, Nigeria; 5 Department of Infectious Diseases, Case Western Reserve University School of Medicine, Cleveland, Ohio, USA; 6 Department of Radiation Biology, Radiotherapy, and Radiodiagnosis, University of Lagos College of Medicine, Lagos, Lagos State, Nigeria; 7 Department of Epidemiology, University of Ilorin, Ilorin, Kwara State, Nigeria; 8 Department of Obstetrics and Gynecology, University of Lagos College of Medicine, Lagos, Lagos State, Nigeria

**Keywords:** Cervical cancer screening, knowledge, Attitude, Practice, Primary healthcare workers, Nigeria.

## Abstract

Abstract

**Background:**

Cervical cancer is a major public health concern globally, particularly in low- and middle-income countries where access to screening and early diagnostic services is limited. Healthcare workers at the Primary Health Care (PHC) level play critical roles in promoting cervical cancer prevention through health education, implementing low-cost cervical cancer screening approaches in low-resource settings, and providing referral services. However, gaps in knowledge and practice among PHC workers may hinder effective cervical cancer control programs.

**Objectives:**

This study assessed knowledge, attitudes, and practices concerning cervical cancer screening among PHC workers in Lagos State, Southwest Nigeria.

**Methods:**

A descriptive cross-sectional study was conducted among 209 PHC workers selected using multistage sampling. Data were collected using a structured questionnaire on socio-demographic characteristics, knowledge of cervical cancer, attitudes toward screening, and screening practices. Data were analysed using descriptive statistics and chi-square tests at a statistical significance level of 5%.

**Results:**

The respondents had a mean age of 36.8 ± 8.8 years. Most participants were female (77.0%) and married (73.7%). Nurses represented the largest professional group (41.1%), followed by community health extension workers (30.6%). Overall, 69% of respondents demonstrated good knowledge of cervical cancer screening, and 89% reported positive attitudes toward screening. However, cervical cancer screening practices were poor, as only 25% of respondents reported actively providing screening services in their facilities. Major barriers included a lack of training, limited screening equipment, and inadequate laboratory support. Significant associations were observed between levels of knowledge and professional category (p < 0.001), educational qualification (p = 0.033 **)**, and years of practice (p = 0.002 **)**.

**Conclusion:**

Strengthening healthcare workers’ capacity, improving infrastructure, and integrating screening into routine PHC services are essential for improving cervical cancer prevention. These findings highlight the need for policy interventions aimed at strengthening primary health care systems in Nigeria in line with global cervical cancer elimination strategies.

## Introduction

Cervical cancer is one of the most preventable forms of cancer, yet it continues to contribute substantially to the global burden of disease among women. Globally, it ranks among the most common cancers affecting women, and it continues as a leading cause of cancer-related deaths in low- and middle-income countries ^1^ The World Health Organization (WHO) rated cervical cancer as the fourth most common cancer among women globally, with more than 600,000 new cases and over 340,000 deaths reported annually. ^2, 3^ The burden of the disease is disproportionately higher in low- and middle-income countries (LMICs), where nearly 90% of cervical cancer deaths occur due to limited access to screening and treatment services. ^1^

Persistent infection with high-risk types of Human Papillomavirus (HPV) is the necessary cause and recognized primary etiological factor in the development of cervical cancer. ^4^ HPV is a common sexually transmitted infection that affects a large proportion of sexually active individuals worldwide. Although most HPV infections resolve spontaneously, persistent infection with oncogenic HPV types can lead to the development of precancerous lesions and eventually invasive cervical cancer if left untreated. ^4– 7^

Cervical cancer is largely preventable through effective primary and secondary prevention strategies. ^8^ Primary prevention involves vaccination against HPV, while secondary prevention focuses on screening and early detection of precancerous lesions. ^8– 10^ The use of screening methods such as Papanicolaou (Pap) smear, liquid-based cytology, HPV DNA testing, and visual inspection with acetic acid (VIA) has been documented to detect early cervical abnormalities before progression to cancer. ^5, 8^ Despite the availability of effective preventive strategies such as HPV vaccination and cervical cancer screening, the burden of the disease remains disproportionately high in sub-Saharan Africa. ^7, 8^

The global health community has increasingly recognized the importance of cervical cancer prevention. By the year 2020, in response to the significant global burden of cervical cancer, the WHO launched the strategic approaches for global cervical cancer elimination as a public health problem, which aim to achieve the “90–70-90” targets. This initiative outlines the “90–70–90” targets to be attained by 2030: achieving HPV vaccination coverage of 90% among girls by age 15, ensuring that 70% of women undergo screening with a high-performance test at least twice (by ages 35 and 45), and providing appropriate treatment for 90% of women diagnosed with cervical precancerous lesions or cancer. ^8, 11^ Achieving these targets requires robust health systems, particularly at the primary healthcare level, and adequately trained healthcare workers who can deliver screening and preventive services in the community. ^11, 12^

Despite these global efforts, cervical cancer remains a major public health issue in many LMICs, including Nigeria. Nigeria has one of the highest cervical cancer burdens in sub-Saharan Africa, with an estimated 12,000 new cases and 8,000 annual deaths. ^13, 14^ Limited access to screening services, inadequate awareness among women, and health system constraints contribute significantly to the persistence of the disease burden. ^7, 15^

Primary Health Care (PHC) systems represent the foundation of health service delivery in many countries. PHC facilities serve as the first point of contact for many individuals within the healthcare system and provide essential services, including health education, disease prevention, early detection, and referral for specialized care. ^16, 17^ In Nigeria, PHC facilities deliver essential community-based health services, including maternal and child health care, immunization, family planning, and health promotion. ^18, 19^Because PHC centers are widely distributed and accessible to communities, they are uniquely positioned to support cervical cancer prevention efforts, particularly in resource-limited settings. ^12, 20^ PHC centres play significant roles in the prevention and control of cervical cancer through community-based health education and promotion, early detection, and referral services. ^8, 11^

PHC workers are key actors in cervical cancer prevention programs. They provide health education on risk factors, symptoms, and preventive strategies. They may perform screening procedures such as visual inspection with acetic acid (VIA) and visual inspection with Lugol’s iodine (VILI), which are recommended low-cost screening methods in low-resource settings. ^11, 21^ They also provide counselling and facilitate referral services for women who require further diagnostic evaluation or treatment at secondary and tertiary healthcare facilities. ^22^

However, studies have reported gaps in knowledge and practice related to cervical cancer screening among healthcare workers in LMICs. Inadequate training, lack of screening equipment, limited laboratory facilities, and weak referral systems have been identified as major barriers to the effective implementation of cervical cancer screening programs. ^23– 26^ In Nigeria, although awareness of cervical cancer screening among healthcare workers has improved, the availability and utilization of screening services remain limited, particularly at the primary healthcare level. ^26– 29^ Previous studies suggest that many PHC facilities lack the infrastructure and trained personnel required to provide screening services, and that healthcare workers’ knowledge and attitudes significantly influence screening uptake and program effectiveness. ^26, 30– 32^

Lagos State, the most populous state in Nigeria, has a complex healthcare system characterized by high population density and substantial demand for health services. ^33^ The state operates a three-tier level of healthcare system with primary, secondary, and tertiary facilities. PHC centers are recognized as the first point of contact for most residents seeking healthcare services. Strengthening cervical cancer screening at this level may therefore yield substantial public health benefits. ^18, 33^

Given the important role of PHC workers in implementing screening services, it is critical to understand their knowledge, attitudes, and practices regarding cervical cancer prevention and improving women’s health outcomes. Identifying existing gaps and barriers to screening implementation can help inform policy decisions and guide targeted interventions aimed at improving screening coverage and reducing cervical cancer morbidity and mortality. Therefore, this study aimed to assess the knowledge, attitudes, and practices, as well as the associated factors, regarding cervical cancer screening among primary healthcare workers in Lagos State, Nigeria. The findings of this study will provide evidence to guide policymakers and healthcare planners in designing strategies to improve cervical cancer screening services within the primary healthcare system.

## Materials and methods

### Study area

This study was conducted in Lagos State, located in the southwestern geopolitical zone of Nigeria. Though the smallest by landmass, Lagos State is the most populous state in Nigeria, with an estimated population exceeding 20 million residents. With Ikeja as its capital city, Lagos State serves as Nigeria’s commercial and financial hub and is characterized by rapid urbanization and socio-economic diversity. Lagos State is divided into 20 officially structured Local Government Areas (LGAs).

Lagos State operates a three-tier healthcare system comprising primary, secondary, and tertiary. ^33^ PHC centres serve as the first point of contact for community health services and are coordinated by the Lagos State Primary Health Care Board under the supervision of the Lagos State Ministry of Health. The state has over 300 PHC centres distributed across the LGAs to provide accessible community-based health services. ^33^ PHC facilities in Lagos State provide cervical cancer prevention services through health promotion and education, community mobilization, and promotion of screening services. They also support HPV vaccination programmes targeting adolescent girls aged 9-14 years and provide referral services for women requiring further diagnostic evaluation or treatment.

### Study design and population

This study used a descriptive cross-sectional design to assess the knowledge, attitudes, and practices of cervical cancer screening among PHC workers in Lagos State, Nigeria. The study population comprised healthcare workers providing services in PHC facilities located within selected LGAs of Lagos State. These included medical doctors, nurses, Community Health Extension Workers (CHEWs), laboratory scientists, and other PHC staff. Male and female PHC workers who must have worked in the PHC setting for at least 6 months were included in the study.

### Sample size determination

The minimum sample size of 156 respondents was calculated using the Cochran formula for descriptive studies: where Z represents the standard normal deviate at a 95% confidence interval (1.96), p represents the estimated proportion of the respondents who knew about cervical cancer screening (89.7%) in a previous study carried out among female healthcare workers in Plateau State, North Central Nigeria, ^34^ q represents 1 − p (10.3%), and d represents the margin of error set at 5%. To account for potential non-participation, an additional 10% was included as a non-response rate in the final calculated minimum sample size.

### Sampling technique

A multistage sampling approach was adopted to select respondents in this study. Initially, in the first stage, three LGAs were selected from the 20 LGAs in Lagos State using simple random sampling through balloting. The selected LGAs were Surulere, Oshodi-Isolo, and Mushin LGAs. Subsequently, in the second stage, three PHCs were selected from each LGA using simple random sampling through balloting. Finally, in the third stage, all clinical PHC workers who were present on duty at the time of data collection were selected and invited to participate in this study.

### Data collection

Data were collected using a pre-tested semi-structured self-administered questionnaire adapted from previous related studies. Five trained research assistants were involved in the data collection. To ensure clarity and reliability, the questionnaire was pre-tested among 20 PHC workers, which was approximately 10% of the sample size, at a PHC in Ikorodu LGA. The questionnaire included sections on socio-demographic characteristics, knowledge of cervical cancer screening, attitudes toward cervical cancer screening, and cervical cancer screening practices.

### Data analysis

Data analysis was conducted using the Statistical Package for Social Sciences (SPSS) version 26. Descriptive statistics were generated and presented as frequencies, percentages, and means with corresponding standard deviations. Respondents’ knowledge, attitudes, and practices regarding cervical cancer screening were categorized and summarized.

Knowledge of cervical cancer screening in terms of eligibility, methods, frequency, treatment options, and the use of the screen and treat algorithm was assessed using 33 questions, with correct responses scored as 1 and incorrect or unknown responses scored as 0. With the maximum obtainable scores of 33 and the minimum obtainable score of zero (0), and using 50% as a cut-off point, scores ranging from 0–16 were classified as poor knowledge of cervical cancer screening, while scores ranging from 17–33 were classified as good knowledge of cervical cancer screening.

Attitudes towards cervical cancer screening were measured using a 5-point Likert scale, with response options scored as strongly agree (5), agree (4), undecided (3), disagree (2), and strongly disagree (1). The attitude section contained 9 scenarios; the total maximum obtainable point was 45, and the total minimum obtainable point was 9. Using 50% as the cut-off, scores ranging from 0–23 were classified as negative attitudes towards cervical cancer screening, while scores ranging from 24–45 were classified as positive attitudes towards cervical cancer screening.

Practice of cervical cancer screening in terms of previous performance of cervical cancer screening on a patient, as well as the referral of the screened and diagnosed cases, was assessed using 6 questions, with correct responses scored as 1 and incorrect or unknown responses scored as 0. With the maximum obtainable scores of 6 and the minimum obtainable score of zero (0), and using 50% as a cut-off point, scores ranging from 0–3 were classified as poor practice of cervical cancer screening, while scores ranging from 4–6 were classified as good practice of cervical cancer screening.

Associations between the sociodemographic characteristics and the knowledge, attitudes, and practices of cervical cancer screening were tested using Chi-square statistics, with statistical significance set at 5% (p ≤ 0.05).

### Ethical considerations

Ethical approval for the study was obtained from the Health Research and Ethics Committee of Lagos University Teaching Hospital (LUTH), with number ADM/DCST/HREC/APP/532. Written informed consent was obtained from all participants before data collection. Participation was entirely voluntary, and confidentiality was ensured by assigning identification numbers to questionnaires in place of personal identifiers. Participants were informed of their right to withdraw at any time without prejudice and provided with contact information for further inquiries or support if needed.

## Results

### Socio-demographic characteristics of respondents

A total of 209 Primary Health Care (PHC) workers participated in the study. The mean age of respondents was 36.8 ± 8.8 years, with the majority aged 30–39 years (40.7%), followed by those 40–49 years (26.8%) and < 30 years (22.0%). Only 10.5% were aged 50 years and above. Females constituted most respondents (77.0%), reflecting the female-dominated nature of the PHC workforce. Most respondents were married (73.7%), while 20.6% were single, and only small proportions were divorced (3.8%) or widowed (1.9%). The dominant ethnic group was Yoruba (77.5%), followed by Igbo (17.2%), reflecting the demographic distribution of Lagos State. Most respondents identified as Christians (80.4%), while 19.6% were Muslims.

Regarding the professional category, nurses constituted the largest group (41.1%), followed by CHEWs (30.6%), medical doctors (16.8%), and laboratory scientists (11.5%). Over half of the respondents (58.4%) held a first degree, while 30.1% had diploma qualifications and 11.5% had postgraduate qualifications. Regarding work experience, 43.5% had practiced for 1–4 years, 27.8% had practiced for over 10 years, and 23.9% had practiced for 5–10 years. Almost half of the respondents (46.4%) earned above ₦100,000 monthly, while 27.3% earned ₦50,000–₦100,000, and 26.3% earned less than ₦50,000.

### Knowledge of cervical cancer screening

Most respondents (72.7%) were aware that screening tests exist for cervical cancer, while 14.4% did not know, and 12.9% reported that no screening test existed. Regarding eligibility for screening, 76.1% correctly identified women aged above 21 years or those sexually active for three years as eligible candidates for screening. However, 63.6% incorrectly believed that all women, irrespective of age and marital status, should undergo screening, indicating some misconceptions regarding screening guidelines.

In terms of screening methods, Pap smear was the most recognized method (85.7%), followed by HPV DNA testing (79.4%), cervical biopsy (79.4%), and visual inspection with acetic acid (VIA) (71.8%). Other methods identified included liquid-based cytology (67.0%), colposcopy (52.6%), and visual inspection with Lugol’s iodine (51.7%). Only 3.4% of respondents incorrectly believed that no screening methods were available. Despite this awareness, 63.2% of respondents reported that cervical cancer screening services were not available in their health facilities, while only 28.7% reported availability of screening services. ( Table 1).

**Table 1.** Respondents’ knowledge of cervical cancer screening.

Variable	Frequency (n = 209)	Percentage (%)
**Knew that there were screening tests for cervical cancer**		
Don’t know	30	14.4
No	27	12.9
Yes	152	72.7
* **People who can undergo cervical cancer screening**		
All females, irrespective of age and marital status	133	63.6
Married women only	9	4.3
Women younger than 21 years of age	71	34.0
Women above 21 years of age or those who are sexually active for the last 3 years	159	76.1
Women above 30 years of age	150	71.8
Only women above 50 years of age	21	10.1
Don’t know	3	1.4
* **Methods of cervical cancer screening you know**		
Pap smear	179	85.7
Human Papillomavirus (HPV) DNA testing	166	79.4
Liquid-based cytology	140	67.0
Visual inspection with acetic acid (VIA)	150	71.8
Visual inspection with Lugol’s solution	108	51.7
Cervical biopsy	166	79.4
Colposcopy	110	52.6
No methods available	7	3.4
Don’t know	7	3.4
**Availability of a cervical cancer screening test in your health facility**		
Don’t know	17	8.1
No	132	63.2
Yes	60	28.7

^*^ Multiple responses were allowed

### Knowledge of screening frequency

Knowledge of the recommended screening frequency for cervical cancer was relatively poor. Nearly 45.5% of respondents incorrectly believed that a Pap smear should be conducted annually, whereas only 14.8% correctly identified the recommended interval of once every three years. Similarly, 33.5% believed HPV DNA testing should be performed annually, while only 9.1% correctly identified the recommended five-year interval. ( Table 2).

**Table 2.** Respondents’ knowledge of frequency of cervical cancer screening.

Variable	Frequency (n = 209)	Percentage (%)
**Frequency of Pap smear test for cervical cancer**		
Don’t know	43	20.6
Every year	95	45.5
Once every 2 years	26	12.4
Once every 3 years	31	14.8
Once every 5 years	5	2.4
Only when symptoms are present	9	4.3
**Frequency of Human Papillomavirus (HPV) DNA testing**		
Don’t know	65	31.1
Every year	70	33.5
Once every 2 years	16	7.7
Once every 3 years	35	16.7
Once every 5 years	19	9.1
Only when symptoms are present	4	1.9

### Knowledge of treatment options

Respondents demonstrated moderate awareness of treatment options following a positive screening result. The most identified treatment option was chemotherapy (79.4%), followed by targeted therapy (61.7%), radiation therapy (59.8%), hysterectomy (56.5%), and cryotherapy (55.5%). However, knowledge of fertility-preserving treatments such as trachelectomy (45.5%) and cold knife cone biopsy (45.0%) was relatively lower. Only 42.1% of respondents had heard of the screen-and-treat algorithm for cervical cancer screening, and among those aware, only 31.8% reported using the approach in their health facilities. ( Table 3).

**Table 3.** Respondents’ knowledge of treatment options for cervical cancer.

Variable	Frequency (n = 209)	Percentage (%)
* **Treatment options for women who test positive after the screening test**		
Hysterectomy (removal of the cervix and uterus)	118	56.5
Cryotherapy	116	55.5
Loop Electrosurgical Excision Procedure (LEEP)	98	46.9
Cold knife cone biopsy (cervical conization)	94	45.0
Trachelectomy (removal of the cervix)	95	45.5
Radiation therapy	125	59.8
Chemotherapy	166	79.4
Targeted drug therapy	129	61.7
Immunotherapy	93	44.5
**Knew of the screen and treat algorithm for cervical cancer screening**		
No	121	57.9
Yes	88	42.1
**Previous use of the screen and treat algorithm for cervical cancer screening in your health facility**	(n = 88)	
No	60	68.2
Yes	28	31.8

^*^ Multiple responses were allowed.

**Figure 1.**
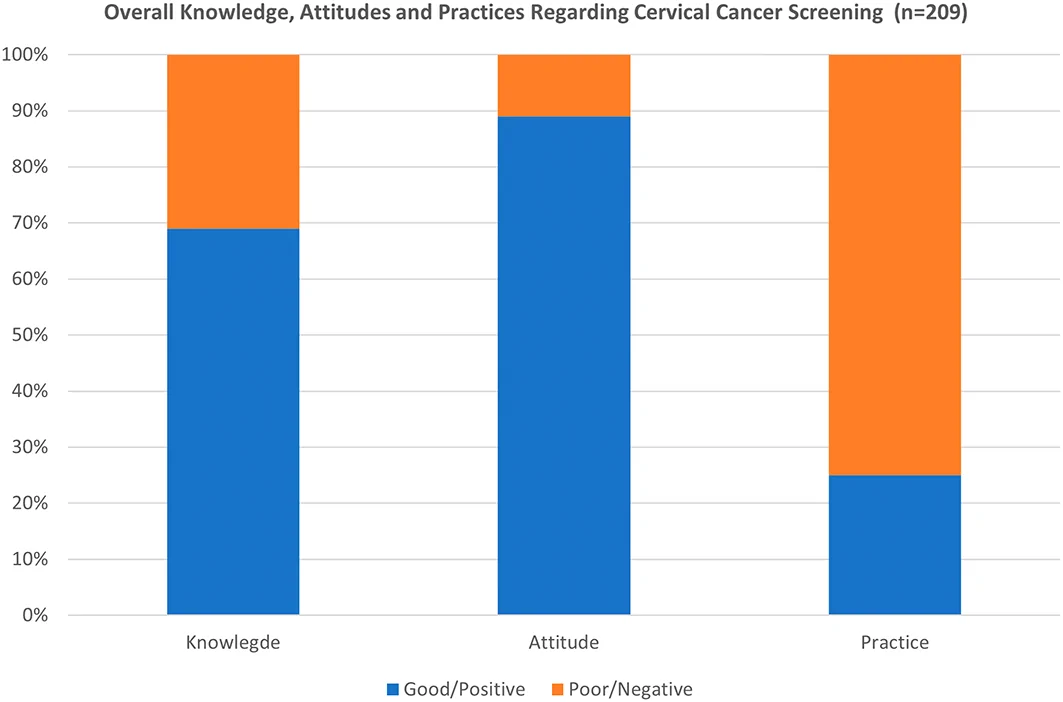
Overall knowledge, attitudes, and practices regarding cervical cancer screening among PHC workers in Lagos State, Nigeria.

Overall, 69% of respondents demonstrated good knowledge of cervical cancer screening, while 31% had poor knowledge. ( Figure 1).

### Attitude towards cervical cancer screening

Most of the respondents showed a positive attitude toward cervical cancer screening. Most respondents (81.8%) strongly agreed that cervical cancer screening is an essential part of women’s health care, and 82.8% strongly agreed that screening facilitates early detection and improved treatment outcomes. Furthermore, 73.2% strongly supported the introduction of cervical cancer screening programs in their communities. About 46.4% strongly agreed that they were willing to perform visual screening of the cervix, while 37.3% agreed with the statement. More than half (54.1%) strongly agreed that additional training was necessary to effectively perform cervical cancer screening, highlighting perceived skill gaps among PHC workers. Only 2.4% strongly agreed that they were too busy to screen women, suggesting that workload was not a major barrier. ( Table 4).

**Table 4.** Attitude of the respondents towards cervical cancer screening.

Variable	Strongly agree n (%)	Agree n (%)	Indifferent n (%)	Disagree n (%)	Strongly disagree n (%)
Cervical cancer screening is an essential part of women’s health care	171 (81.8)	34 (16.3)	3 (1.4)	0 (0.0)	1 (0.5)
I am willing to do a visual screening of the cervix on my patients	97 (46.4)	78 (37.3)	29 (13.9)	2 (1.0)	3 (1.4)
Cervical cancer screening is too difficult	17 (8.1)	25 (12.0)	24 (11.5)	85 (40.7)	58 (27.7)
Cervical cancer screening canhelp in early detection and better treatment	173 (82.8)	27 (12.9)	5 (2.4)	3 (1.4)	1 (0.5)
A cervical cancer screening program should start in my community	153 (73.2)	50 (23.9)	4 (1.9)	2 (1.0)	0 (0.0)
Cervical cancer screening is too expensive	25 (12.0)	44 (21.0)	50 (23.9)	58 (27.8)	32 (15.3)
My patients have more important health issues than cervical cancer screening	16 (7.7)	27 (12.9)	41 (19.6)	59 (28.2)	66 (31.6)
I need additional training to successfully perform cervical cancer screening	113 (54.1)	77 (36.8)	7 (3.4)	9 (4.3)	3 (1.4)
I am too busy to screen women for cervical cancer	5 (2.4)	14 (6.7)	29 (13.9)	92 (44.0)	69 (33.0)

Overall, 89% of respondents demonstrated a positive attitude toward cervical cancer screening, while 11% showed a negative attitude. ( Figure 1).

### Practice of cervical cancer screening

Despite relatively good knowledge and positive attitudes, screening practices were poor. Only 24.9% of respondents had ever performed cervical cancer screening on a patient, while 75.1% had never conducted a screening test. Among those who had performed screening, visual inspection with acetic acid (VIA) was the most used method (67.3%), followed by Pap smear (50.0%), colposcopy (23.1%), VILI (19.2%), and liquid-based cytology (19.2%). Only 3.9% had performed HPV DNA testing. ( Table 5).

**Table 5.** Respondents’ performance of cervical cancer screening.

Variable	Frequency (n = 209)	Percentage (%)
**Performed cervical cancer screening on a patient before**		
No	157	75.1
Yes	52	24.9
* **Ifyes,typeofcervicalcancerscreening performed**	**(n = 52)**	
Visual inspection of the cervix with acetic acid (VIA)	35	67.3
Visual inspection of the cervix with lugol’s iodine (VILI)	10	19.2
Pap smear test using Ayre’s spatula	26	50.0
Human Papillomavirus (HPV) DNA testing	2	3.9
Liquid-based cytology	10	19.2
Cervical biopsy	8	15.4
Colposcopy	12	23.1
**Number of times you have performed cervical cancer screening on a patient**		
1–5	39	18.7
6–10	4	1.9
>10	11	5.3
Never	155	74.2
**`** * **If you have never performed cervical cancer screening on a patient before, why**	**(n = 155)**	
Absence of indication	29	18.7
Lack of necessary equipment or supplies needed	61	39.4
Lack of necessary laboratory resources to screen	64	41.3
I do not have the necessary training to screen a patient for cervical cancer	98	63.2
Cervical cancer screening is a doctor’s procedure	120	77.4

^*^ Multiple responses were allowed

### Screening practices

Screening experience was also limited among all the respondents, as only 5.3% had performed screening more than ten times, while 74.2% had never conducted any screening. The major barriers to screening among those who had never conducted cervical cancer screening on a patient included lack of training (63.2%), lack of laboratory resources (41.3%), and lack of equipment or supplies (39.4%). ( Table 5).

### Referral practices

About 75.6% of respondents reported referring patients or acquaintances for cervical cancer screening, although only 45.0% routinely asked patients whether they had previously undergone screening. Approximately 25.4% of respondents had diagnosed pre-cancerous cervical lesions, and most referred these patients to general hospitals (50.9%) or teaching hospitals (20.8%). Similarly, 20.6% had diagnosed cervical cancer, and 27.9% referred these patients to gynaecologists in hospitals. ( Table 6).

**Table 6.** Respondents’ practice of referrals concerning cervical cancer screening.

Variable	Frequency (n = 209)	Percentage (%)
**Encouraged and/or referred some friends and relations/patients to present themselves for cervical cancer screening**		
No	51	24.4
Yes	158	75.6
**Routinely ask patients if they have been screened for cervical cancer**		
No	115	55.0
Yes	94	45.0
**If yes, what is the age group of patients you ask (years)**	**(n = 94)**	
<20	1	1.1
20–30	48	51.1
30–40	36	38.3
40–50	7	7.4
>50	2	2.1
**Previously diagnosed pre-cancerous cervical lesions in a patient**		
No	156	74.6
Yes	53	25.4
**If yes, what did you do?**	**(n = 53)**	
Cervical Biopsy	15	28.3
Referred to General Hospital	27	50.9
Referred to the Teaching Hospital	11	20.8
**Previously diagnosed a patient with cervical cancer**		
No	166	79.4
Yes	43	20.6
**If yes, what did you do?**	**(n = 43)**	
Carried out a pap smear test	17	39.5
Cryotherapy	4	9.3
Referred to a Gynecologist in the General Hospital	12	27.9
Visual inspection with acetic acid (VIA)	10	23.3
**Where do you refer patients to if there is a need for referral**		
General Hospital	24	55.8
Teaching Hospital	16	37.2
Oncologist	3	7.0
**Interpreted the result of pap smear test to patients requiring such services**		
No	151	72.2
Yes	58	27.8

Overall, 75% of respondents demonstrated poor screening practice, while 25% demonstrated good practice. ( Figure 1).

### Factors associated with knowledge, attitude, and practice of cervical cancer screening

Knowledge of cervical cancer screening was statistically significantly associated with professional category ( *P* < 0.001), level of qualification ( *P* = 0.033), duration of practice ( *P* = 0.002), and income ( *P* < 0.001). Medical doctors (91.4%) and nurses (76.7%) demonstrated higher levels of knowledge compared with CHEWs (54.7%) and laboratory scientists (45.8%). However, there was no statistically significant association between the overall knowledge of cervical cancer screening and age, sex, marital status, ethnicity, or religion ( *P* > 0.05). Attitudes toward cervical cancer screening were generally favourable across all socio-demographic groups, with no significant associations observed except for duration of practice ( *P* = 0.021) and income ( *P* = 0.033). In contrast, cervical cancer screening practice was significantly associated with multiple variables, including marital status ( *P* = 0.009), ethnicity ( *P* = 0.002), religion ( *P* = 0.043), professional category ( *P* < 0.001), qualification ( *P* = 0.015), duration of practice ( *P* < 0.001), and income ( *P* < 0.001). Good practice of cervical cancer screening was highest among medical doctors (54.3%) and lowest among laboratory scientists (4.2%). ( Table 7).

**Table 7.** Association between socio-demographic characteristics and knowledge, attitude, and practice of cervical cancer screening among phc workers in lagos state.

Variables	Knowledge		Attitude		Practice	
	Good	Poor	Positive	Negative	Good	Poor
	n = 144	n = 65	n = 187	n = 22	n = 52	n = 157
	n (%)	n (%)	n (%)	n (%)	n (%)	n (%)
**Age group**						
<30	28 (60.87)	18 (39.13)	41 (89.13)	5 (10.87)	9 (19.57)	37 (80.43)
30–39	62 (72.94)	23 (27.06)	75 (88.24)	10 (11.76)	20 (23.53)	65 (76.47)
40–49	39 (69.64)	17 (30.36)	52 (92.86)	4 (7.14)	14 (25.00)	42 (75.00)
≥50	15 (68.18)	7 (31.82)	19 (86.36)	3 (13.64)	9 (40.91)	13 (59.09)
	χ2 = 2.05	p = 0.562	χ2 = 1.05	p = 0.741	χ2 = 3.80	p = 0.284
**Sex**						
Female	106 (65.84)	55 (34.16)	144 (89.44)	17 (10.56)	36 (22.36)	125 (77.64)
Male	38 (79.17)	10 (20.83)	43 (89.58)	5 (10.42)	16 (33.33)	32 (66.67)
	χ2 = 0.08	p = 0.109	χ2 = 0.98 ^**a**^	p = 1.000	χ2 = 0.12	p = 0.132
**Marital status**						
Single, never married	25 (58.14)	18 (41.86)	37 (86.05)	6 (13.95)	10 (23.26)	33 (76.74)
Married	108 (70.13)	46 (29.87)	139 (90.26)	15 (9.74)	34 (22.08)	120 (77.92)
Divorced	7 (87.50)	1 (12.50)	7 (87.50)	1 (12.50)	5 (62.50)	3 (37.50)
Widowed	4 (100.00)	0 (0.00)	4 (100.00)	0 (0.00)	3 (75.00)	1 (25.00)
	χ2 = 5.53 ^**a**^	p = 0.171	χ2 = 1.14 ^**a**^	p = 0.704	χ2 = 12.14 ^**a**^	**p = 0.009** ^*^
**Ethnicity**						
Yoruba	113 (69.75)	49 (30.25)	146 (90.12)	16 (9.88)	45 (27.78)	117 (72.22)
Igbo	22 (61.11)	14 (38.89)	30 (83.33)	6 (16.67)	2 (5.56)	34 (94.44)
Hausa	1 (50.00)	1 (50.00)	2 (100.00)	0 (0.00)	0 (0.00)	2 (100.00)
Others	8 (88.89)	1 (11.11)	9 (100.00)	0 (0.00)	5 (55.56)	4 (44.44)
	χ2 = 3.09 ^**a**^	p = 0.333	χ2 = 2.81 ^**a**^	p = 0.470	χ2 = 13.11 ^**a**^	p **= 0.002** ^*^
**Religion**						
Christianity	113 (67.26)	55 (32.74)	153 (91.07)	15 (8.93)	47 (27.98)	121 (72.02)
Islam	31 (75.61)	10 (24.39)	3 (82.93)	7 (17.07)	5 (12.20)	36 (87.80)
	χ2 = 3.30 ^**a**^	p = 0.350	χ2 = 0.13 ^**a**^	p = 0.154	χ2 = 0.04 ^**a**^	p **= 0.043** ^*^
**Professional category**						
CHEWS * *	35 (54.69)	29 (45.31)	54 (84.38)	10 (15.63)	8 (12.50)	56 (87.50)
Laboratory scientists	11 (45.83)	13 (54.17)	22 (91.67)	2 (8.33)	1 (4.17)	23 (95.83)
Medical doctor	32 (91.43)	3 (8.57)	35 (100.00)	0 (0.00)	19 (54.29)	16 (45.71)
Nurses	66 (76.74)	20 (23.26)	76 (88.37)	10 (11.63)	24 (27.91)	62 (72.09)
	χ2 = 22.75	**p < 0.001** ^*^	χ2 = 6.12 ^**a**^	p = 0.063	χ2 = 27.37	**p < 0.001** ^*^
**Level of qualification**						
First Degree (BSc, MBBS)	88 (72.13)	34 (27.87)	113 (92.62)	9 (7.38)	31 (25.41)	91 (74.59)
Diploma	36 (57.14)	27 (42.86)	52 (82.54)	11 (17.46)	10 (15.87)	53 (84.13)
Postgraduate	20 (83.33)	4 (16.67)	22 (91.67)	2 (8.33)	11 (45.83)	13 (54.17)
	χ2 = 6.99	**p = 0.033** ^*^	χ2 = 4.62	p = 0.111	χ2 = 8.39	**p = 0.015** ^*^
**Duration of practice (years)**						
< 1	3 (30.00)	7 (70.00)	6 (60.00)	4 (40.00)	0 (0.00)	10 (100.00)
1–4	59 (64.84)	32 (35.16)	81 (89.01)	10 (10.99)	10 (10.99)	81 (89.01)
5–10	43 (86.00)	7 (14.00)	48 (96.00)	2 (4.00)	24 (48.00)	26 (52.00)
> 10	39 (67.24)	19 (32.76)	52 (89.66)	6 (10.34)	18 (31.03)	40 (68.97)
	χ2 = 14.66	**p = 0.002** ^*^	χ2 = 11.50	**p = 0.021** ^*^	χ2 = 28.18	**p < 0.001** ^*^
**Income (Naira)**						
0 - < 49,999	24 (43.64)	31 (56.36)	44 (80.00)	11 (20.00)	3 (5.45)	52 (94.55)
50,000-100,000	40 (70.18)	17 (29.82)	52 (91.23)	5 (8.77)	5 (8.77)	52 (91.23)
>100,000	80 (82.47)	17 (17.53)	91 (93.81)	6 (6.19)	44 (45.36)	53 (54.64)
	χ2 = 24.76	**p < 0.001** ^*^	χ2 = 7.37	**p = 0.033** ^*^	χ2 = 40.79	**p < 0.001** ^*^

Note: χ2 - Chi square Test,

Abbreviations: CHEW - Community Health Extension Workers; BSc - Bachelor of Science; MBBS - Bachelor of Medicine, Bachelor of Surgery.

^a^ Fisher’s exact test, p - p-value,

^*^ Statistically significant at p < 0.05,

^**^ CHEW.

## Discussion

This study assessed knowledge, attitudes, and practices regarding cervical cancer screening among PHC workers in Lagos State, Nigeria. The findings reveal that although most respondents demonstrated good knowledge (69%) and positive attitudes (89%) toward cervical cancer screening, the actual practice of screening services was poor (25%). This discrepancy highlights an important implementation gap between awareness and service delivery within the primary health care system, and it reflects broader challenges affecting cervical cancer prevention programs in many LMICs.

### Knowledge of cervical cancer screening

The study found that a majority of PHC workers had good knowledge of cervical cancer screening, which is encouraging given the critical role of healthcare workers in promoting preventive health services. Similar levels of knowledge have been reported among healthcare providers in other LMICs. For example, studies conducted in Ethiopia, Cameroon, and Uganda reported that most healthcare workers were aware of cervical cancer and recognized screening as an important preventive strategy. ^35– 37^ The high level of awareness may be attributed to the increasing global attention to cervical cancer prevention, professional training, and exposure to health education campaigns.

However, this study identified important knowledge gaps regarding screening eligibility and screening intervals. A significant proportion of respondents incorrectly believed that cervical cancer screening (including Pap smear and HPV DNA testing) should be conducted annually. Current international guidelines recommend cytology screening every three years for women aged 21–65 years and HPV DNA testing every five years in appropriate populations. ^8, 11, 38^ Similar misconceptions regarding screening intervals have been reported among healthcare workers in Nigeria, Ethiopia, Tanzania, and South Africa. ^39– 42^ This may reflect inadequate familiarity with current international guidelines. Poor knowledge of screening intervals may contribute to inconsistent screening practices and inappropriate clinical recommendations to patients.

The study also demonstrates limited awareness of the screen-and-treat approach, with fewer than half of respondents familiar with this strategy. The screen-and-treat model is recommended by WHO for low-resource settings, as it enables same-day treatment following screening and minimises loss to follow-up. ^8, 20, 43, 44^ Limited awareness of this strategy among PHC workers suggests a critical need for targeted training and guideline dissemination.

### Attitudes toward cervical cancer screening

The study also found that most respondents had positive attitudes toward cervical cancer screening, with most recognizing screening as an essential component of women’s health care and its importance for early detection and improved treatment outcomes. Most respondents strongly supported the implementation of screening programs in their communities. Positive attitudes among healthcare providers have been widely reported in previous studies. For instance, research conducted in Uganda and Ethiopia found that healthcare workers generally support cervical cancer prevention programs and recognize the importance of early detection. ^37, 45^

Healthcare workers are key influencers of patient behaviour, and positive provider attitudes are critical for improving cervical cancer screening uptake among women. Existing evidence indicates that a healthcare provider’s recommendation is one of the strongest determinants of uptake of cervical cancer screening services. ^46– 48^ When healthcare providers actively promote screening, women are significantly more likely to undergo screening tests. Consequently, healthcare workers’ attitudes toward screening play a crucial role in improving screening uptake. Despite these positive attitudes, many respondents in this study reported needing additional training to perform cervical cancer screening effectively. Similar findings have been reported in studies conducted in Nigeria, Kenya, and other LMICs, where healthcare workers expressed willingness to provide screening services but lacked adequate training or confidence in their clinical skills. ^23, 26, 49, 50^

### Cervical cancer screening practices among PHC workers

Although respondents demonstrated reasonable knowledge levels and positive attitudes, screening practices were poor, with 75% demonstrating poor screening practices overall. Only about one-quarter of respondents reported having performed cervical cancer screening. The discrepancy between knowledge and actual practice of cervical cancer screening has been reported in several studies among healthcare providers in LMICs. For example, studies conducted among healthcare workers in Ethiopia and South Africa found that although most respondents were aware of cervical cancer screening, only a small proportion had ever performed screening procedures. ^40, 42, 51, 52^ Similar research in Nigeria found that healthcare workers often had adequate knowledge but rarely performed screening due to structural and institutional barriers. ^22, 29^

Several factors may explain the low screening practice observed in this study. The most reported barrier was a lack of training, with many respondents indicating that they lacked the necessary skills to perform screening procedures. This barrier has also been documented in studies from Ethiopia. ^40, 51^ Healthcare worker training has been identified as a key determinant of cervical cancer screening implementation in many LMICs; thus, the training gap among healthcare workers is a major barrier to cervical cancer screening implementation, especially in LMICs. ^53, 54^ In addition, inadequate availability of screening equipment and limited laboratory capacity and resources emerged as key barriers. Many respondents reported that screening services were unavailable in their facilities due to inadequate infrastructure and resources. Similar barriers have been documented in a systematic review focused on sub-Saharan African countries, where limited resources and infrastructure significantly constrained screening programs. ^30^ These barriers are commonly reported in low-resource health systems and often limit the integration of cervical cancer screening into routine PHC services. In addition, healthcare workers may perceive cervical cancer screening as a specialized procedure performed primarily by gynaecologists, which may discourage PHC workers from providing screening services. However, global health strategies increasingly emphasize task-shifting and task-sharing approaches, where trained nurses and community health workers provide appropriate screening services at primary healthcare facilities. ^8, 55^

Among respondents who had performed screening, visual inspection with acetic acid (VIA) was the most used screening method. This finding aligns with global recommendations for cervical cancer screening in resource-constrained settings. VIA is widely recommended for resource-limited settings because it is low-cost, easy to perform, and provides immediate results, making it suitable for use in PHC facilities without sophisticated laboratory infrastructure. ^21, 56^ Several countries in Africa and Asia have successfully implemented VIA-based screening programs at the primary healthcare level as part of national cervical cancer prevention strategies.

However, the study found extremely low utilization of HPV DNA testing, which reflects the limited availability of advanced diagnostic technologies in primary healthcare settings. Although HPV testing is increasingly recommended as the primary screening method in many countries, its implementation in LMICs remains limited and challenging due to high costs and laboratory requirements. ^7, 8, 11^ Expanding access to affordable HPV testing technologies may improve future screening coverage.

The study found that most respondents reported referring patients for cervical cancer screening or treatment, indicating some level of engagement with cervical cancer prevention efforts. Referral systems and practices are particularly important in PHC settings, where diagnostic and treatment services may be unavailable. The referral practices observed in this study suggest that PHC workers recognize the importance of specialist care for suspected cervical cancer cases.

However, fewer than half of respondents routinely asked patients about their cervical cancer screening history. This suggests missed opportunities for opportunistic screening for preventive care during routine consultations, which has been recommended as an effective strategy for increasing screening coverage in low-resource settings. ^27, 57^ Integrating cervical cancer screening into maternal and reproductive health services, including antenatal care and family planning clinics, has been shown to significantly improve screening uptake. ^58^ PHC workers are uniquely positioned to deliver these integrated services because they interact frequently with women of reproductive age.

### Factors associated with knowledge and practice of cervical cancer screening

This study demonstrates that knowledge of cervical cancer screening among PHC workers was not influenced by basic socio-demographic characteristics such as age, sex, marital status, ethnicity, or religion. This suggests that exposure to information on cervical cancer may be relatively widespread across these groups. However, knowledge was significantly associated with professional and socioeconomic factors, including professional category, level of qualification, duration of practice, and income. These findings indicate that formal training, clinical exposure, and professional advancement are critical in shaping PHC workers’ knowledge and skills in cervical cancer prevention.

Medical doctors and nurses demonstrated significantly higher levels of knowledge compared with CHEWs and laboratory scientists. These findings are consistent with studies conducted in South Africa, Tanzania, Somalia, and Ethiopia, where higher professional qualifications and training, as well as longer clinical experience, were associated with better knowledge of cervical cancer prevention and participation in screening programs. ^31, 41, 51, 59^ This pattern underscores the influence of educational background and professional exposure on knowledge acquisition among healthcare workers. Given that CHEWs and nurses often serve as frontline providers in PHC settings and are the first point of contact for many women, strengthening their capacity through targeted, continuous professional development training programs is essential to improving cervical cancer prevention outcomes.

Attitudes toward cervical cancer screening were generally favourable across socio-demographic groups. This suggests that while willingness to support screening is high, it is not necessarily dependent on professional category or background characteristics. Similar findings have been reported in sub-Saharan Africa, where healthcare workers often demonstrate positive attitudes despite gaps in knowledge and practice. ^45^ The minimal variation with significant differences observed in duration of practice and income indicates that higher years of professional experience and socio-economic status may enhance a positive attitude to cervical cancer screening.

In contrast, screening practice was significantly associated with multiple socio-demographic and professional variables, highlighting substantial inequities in service delivery. The markedly higher practice levels among medical doctors compared with CHEWs and laboratory scientists underscore the influence of clinical training and role expectations. These findings reflect persistent structural and capacity-related barriers, including limited skills, inadequate training, and restricted access to screening resources at the PHC level. Evidence from WHO guidelines emphasizes the importance of task-shifting and targeted capacity building to enable lower-cadre health workers to deliver screening services. ^8^ Addressing these disparities through continuous professional training and health system strengthening is essential to improve screening uptake and achieve equitable cervical cancer prevention outcomes.

### Strengths and limitations of the study

This study had several strengths. First, it assessed multiple dimensions of cervical cancer screening, including knowledge, attitude, and practice among PHC workers. Second, the study included various categories of healthcare professionals, allowing for comparison across professional groups. Third, the use of structured questionnaires and standardized data analysis improved the reliability of the findings. However, certain limitations should be acknowledged when interpreting the results. First, the cross-sectional design limits the ability to establish causal relationships between variables. Second, reliance on self-reported information introduces the possibility of recall bias and social desirability biases. Third, the primary focus of this study was on PHC facilities within Lagos State, which may limit the extent to which the findings can be generalized to other regions of Nigeria. Despite these limitations, the study provides valuable insights into the knowledge gaps, training needs, and structural barriers affecting cervical cancer screening services in primary healthcare settings.

## Conclusion

This study assessed the knowledge, attitudes, and practices of cervical cancer screening among primary healthcare workers in Lagos State, Nigeria. The findings revealed that although most healthcare workers demonstrated good knowledge and positive attitudes toward cervical cancer screening, the actual implementation of screening services remains limited. Only a small proportion of respondents reported performing screening procedures in their facilities, indicating a significant gap between awareness and practice.

The study also identified key barriers to cervical cancer screening at the primary healthcare level, including inadequate training, lack of screening equipment, and limited laboratory infrastructure. These challenges may hinder the effective implementation of cervical cancer prevention programs and contribute to the continued burden of the disease. Strengthening primary healthcare workers’ capacity through regular training programs and improving the availability of screening equipment and resources are essential for expanding cervical cancer screening services. Additionally, integrating cervical cancer screening into routine maternal and reproductive health services may help improve access and uptake among women.

### Recommendations

The findings of this study have several important implications for strengthening cervical cancer prevention programs through the PHC systems in Nigeria and similar LMICs to achieve the global targets of eliminating cervical cancer as a public health problem by 2030. First, capacity building for PHC workers is essential. Regular training programs should be implemented to improve healthcare workers’ knowledge and skills in cervical cancer screening techniques such as VIA and HPV testing. Second, screening services should be integrated into routine PHC services, particularly maternal and reproductive health programs, to improve early detection and prevention of cervical cancer. Task-shifting strategies that enable trained nurses and community health workers to perform screening procedures may also help expand screening coverage. Third, health system strengthening is necessary to ensure that PHC facilities are adequately equipped with screening equipment and supplies. Government investment in screening infrastructure will be essential for expanding cervical cancer prevention services. Fourth, strengthening referral systems between primary, secondary, and tertiary healthcare facilities. PHC workers should also be trained on appropriate referral services. Fifth, enhancement of community awareness programs to promote prevention and early screening. PHC workers should continue to sensitize the community about cervical cancer prevention. Sixth, further studies should explore the effectiveness of community-based screening interventions and evaluate strategies for improving HPV vaccination and screening coverage in Nigeria. Finally, improving cervical cancer prevention requires strong policy support and national cervical cancer control programs aligned with the WHO cervical cancer elimination strategy, which emphasizes vaccination, screening, and treatment as key pillars of cervical cancer prevention. Addressing these gaps will be critical for achieving the global cervical cancer elimination targets by 2030, which will depend on strengthening primary healthcare systems and empowering frontline healthcare workers to provide preventive services.

## Data Availability

Repository name: Zenodo: Gaps in Knowledge and Practice of Cervical Cancer Screening among Primary Health Care Workers in Lagos, Nigeria. https://doi.org/10.5281/zenodo.19609449. ^60^ The project contains the following underlying data:
• Data.xls (Raw data used for statistical analysis). Data are available under the terms of the Creative Commons Attribution 4.0 International license (CC-BY 4.0). Repository name: Zenodo: Gaps in Knowledge and Practice of Cervical Cancer Screening among Primary Health Care Workers in Lagos, Nigeria. https://doi.org/10.5281/zenodo.19609449. ^60^ This project contains the following extended data:
• Informed Consent Form and Questionnaire.pdf (Survey instrument used for data collection). Data are available under the terms of the Creative Commons Attribution 4.0 International license (CC-BY 4.0).

## References

[1] Arbyn M Weiderpass E Bruni L : Estimates of incidence and mortality of cervical cancer in 2018: a worldwide analysis. *Lancet Glob Health.* 2020 Feb;8(2):e191–e203. 10.1016/S2214-109X(19)30482-6 31812369 PMC7025157

[2] Ferlay J Colombet M Soerjomataram I : Cancer statistics for the year 2020: An overview. *Int J Cancer.* 2021 Apr 5;149:778–789. 10.1002/ijc.33588 33818764

[3] Bray F Ferlay J Soerjomataram I : Global cancer statistics 2018: GLOBOCAN estimates of incidence and mortality worldwide for 36 cancers in 185 countries. *CA Cancer J Clin.* 2018 Nov;68(6):394–424. 10.3322/caac.21492 30207593

[4] Walboomers JM Jacobs MV Manos MM : Human papillomavirus is a necessary cause of invasive cervical cancer worldwide. *J Pathol.* 1999 Sep;189(1):12–19. 10.1002/(SICI)1096-9896(199909)189:1<12::AID-PATH431>3.0.CO;2-F 10451482

[5] Schiffman M Castle PE Jeronimo J : Human papillomavirus and cervical cancer. *Lancet.* 2007 Sep 8;370(9590):890–907. 10.1016/S0140-6736(07)61416-0 17826171

[6] Bosch FX Burchell AN Schiffman M : Epidemiology and natural history of human papillomavirus infections and type-specific implications in cervical neoplasia. *Vaccine.* 2008 Aug 19;26 Suppl 10:K1–K16. 10.1016/j.vaccine.2008.05.064 18847553

[7] Bruni L Albero G Serrano B : ICO/IARC Information Centre on HPV and Cancer (HPV Information Centre). *Human Papillomavirus and Related Diseases in the World. Summary Report.* 2023 Mar. Reference Source

[8] World Health Organization: *Global strategy to accelerate the elimination of cervical cancer as a public health problem.* Geneva: WHO;2020.

[9] WHO: *Tracking NCDs: best buy and other recommended interventions for the prevention and control of non-communicable diseases.* 2nd ed. 2024.

[10] WHO: Vaccine in National Immunization Programme Update. 2020.

[11] World Health Organization: *WHO guidelines for screening and treatment of precancerous lesions for cervical cancer prevention.* Geneva: WHO;2021. 24716265

[12] World Health Organization: *Primary health care: transforming vision into action.* Geneva: WHO;2018.

[13] Sung H Ferlay J Siegel RL : Global cancer statistics 2020: globocan estimates of incidence and mortality worldwide for 36 cancers in 185 countries. *CA Cancer J Clin.* 2021 May;71(3):209–249. 10.3322/caac.21660 33538338

[14] Ferlay J Ervik M Lam F : *Global Cancer Observatory: Cancer Today - Nigeria.* Lyon, France:2024 [cited 2026 Mar 17]. Reference Source

[15] Ezechi OC Gab-Okafor CV Ostergren PO : Willingness and acceptability of cervical cancer screening among HIV positive Nigerian women. *BMC Public Health.* 2013 Jan 17;13:46. 10.1186/1471-2458-13-46 23327453 PMC3567931

[16] Starfield B : *Primary care: balancing health needs, services, and technology.* New York: Oxford University Press;1998. 10.1093/oso/9780195125429.001.0001

[17] World Health Organization: *The World Health Report 2008. Primary Health Care: Now More Than Ever.* Geneva:2008. 10.30875/e89f8212-en

[18] National Primary Health Care Development Agency: *National Primary Health Care Policy.* Abuja: NPHCDA;2016.

[19] National Primary Health Care Development Agency: *Minimum Standard for Primary Health Care in Nigeria.* Abuja: NPHCDA;2012.

[20] Denny L Sanjose S de Mutebi M : Interventions to close the divide for women with breast and cervical cancer between low-income and middle-income countries and high-income countries. *Lancet.* 2017 Feb 25;389(10071):861–870. 10.1016/S0140-6736(16)31795-0 27814963

[21] Sankaranarayanan R Nessa A Esmy PO : Visual inspection methods for cervical cancer prevention. *Best Pract Res Clin Obstet Gynaecol.* 2012 Apr;26(2):221–232. 10.1016/j.bpobgyn.2011.08.003 22075441

[22] Onyenwenyi AOC Mchunu GG : Primary health care workers’ understanding and skills related to cervical cancer prevention in Sango PHC centre in south-western Nigeria: a qualitative study. *Prim Health Care Res Dev.* 2019 Jul 1;20:e93. 10.1017/S1463423619000215 32799996 PMC6609971

[23] Petersen Z Jaca A Ginindza TG : Barriers to uptake of cervical cancer screening services in low-and-middle-income countries: a systematic review. *BMC Womens Health.* 2022 Dec 2;22(1):486. 10.1186/s12905-022-02043-y 36461001 PMC9716693

[24] Rajendra Datey DrA: Assessment of facility readiness and knowledge gaps among service providers for cervical cancer screening at the primary health care level. *African Journal of Biomedical Research.* 2024 Sep 30;927–934. 10.53555/ajbr.v27i3s.2164

[25] Nyaaba JA Akurugu E : Knowledge, barriers and uptake towards Cervical Cancer screening among female health workers in Ghana: A perspective of the Health Belief Model. *Int J Afr Nurs Sci.* 2023 Jan 19;100587. 10.1016/j.ijans.2023.100587

[26] Irowa O Adeyeye OS Ujah OI : Assessment of barriers to cervical cancer screening at primary health care centers in Makurdi, North-Central Nigeria: a mixed-methods study. *BMC Cancer.* 2025 Jul 1;25(1):1099. 10.1186/s12885-025-14494-1 40598064 PMC12211423

[27] Okolie EA Aluga D Anjorin S : Addressing missed opportunities for cervical cancer screening in Nigeria: a nursing workforce approach. *Ecancermedicalscience.* 2022;16. 10.3332/ecancer.2022.1373 35702415 PMC9116993

[28] Tobin EA Ifada SO Obi AI : Cervical cancer screening risk perception, uptake, and associated factors among female primary healthcare workers in edo state, nigeria. *International Journal of Medicine and Health Development.* 2024;29(1):17–27. 10.4103/ijmh.ijmh_41_23

[29] Olubodun T Olaniran A Awowole IO : Practice of opportunistic cervical cancer screening and health education among health workers in Ogun State, nigeria: A qualitative study of barriers and facilitators. *PLoS One.* 2025 Sep 1;20(9 September):e0316883. 10.1371/journal.pone.0316883 40934253 PMC12425320

[30] Sichalwe MM Basit A Rehman AU : Healthcare infrastructure and cervical cancer screening in low-resource settings: A systematic review focused on Sub-Saharan Africa. *SSM - Health Systems.* 2026 Jun;6:100186. 10.1016/j.ssmhs.2026.100186

[31] Ncane Z Gonah L Estrada GAP : Knowledge, attitudes, and practices of healthcare workers on cervical cancer screening in rural healthcare facilities of the eastern cape. *Healthcare (Switzerland).* 2025 Sep 1;13(18). 10.3390/healthcare13182316 41008446 PMC12469757

[32] Oche MO Kaoje UM Gana G : Cancer of the cervix and cervical screening: Current knowledge, attitude and practices of female health workers in sokoto, nigeria. *International Journal of Medicine and Medical Sciences.* 2013;5(3):106–109. 10.5897/IJMMS12.055

[33] Lagos State Ministry of Health: *Health sector strategic plan.* Lagos: LSMOH;2020.

[34] Utoo PM Utoo B : Knowledge, practices and education of clients on cervical cancer screening among female health care workers in plateau state, nigeria. *Nigerian Medical Journal.* 2011;52(2):119–122.

[35] Dulla D Daka D Wakgari N : Knowledge about cervical cancer screening and its practice among female health care workers in Southern Ethiopia: A cross-sectional study. *Int J Womens Health.* 2017 May 22;9:365–372. 10.2147/IJWH.S132202 28579837 PMC5446960

[36] McCarey C Pirek D Tebeu PM : Awareness of HPV and cervical cancer prevention among Cameroonian healthcare workers. *BMC Womens Health.* 2011 Oct 18;11:45. 10.1186/1472-6874-11-45 22008186 PMC3219551

[37] Mutyaba T Mmiro FA Weiderpass E : Knowledge, attitudes and practices on cervical cancer screening among the medical workers of Mulago Hospital, Uganda. *BMC Med Educ.* 2006 Mar 1;6:13. 10.1186/1472-6920-6-13 16509979 PMC1413529

[38] US Preventive Services Task Force Curry SJ Krist AH : Screening for Cervical Cancer: US Preventive Services Task Force Recommendation Statement. *JAMA.* 2018 Aug 21;320(7):674–686. 10.1001/jama.2018.10897 30140884

[39] Uchendu I Hewitt-Taylor J Turner-Wilson A : *Knowledge, attitudes, and perceptions about cervical cancer, and the uptake of cervical cancer screening in Nigeria: An integrative review. Scientific African.* Elsevier B.V.;2021. 10.1016/j.sciaf.2021.e01013

[40] Abebaw E Tesfa M Gezimu W : Female healthcare providers’ knowledge, attitude, and practice towards cervical cancer screening and associated factors in public hospitals of Northwest Ethiopia. *SAGE Open Med.* 2022;10:20503121221095932. 10.1177/20503121221095931 35600715 PMC9118899

[41] Urasa M Darj E : Knowledge of cervical cancer and screening practices of nurses at a regional hospital in Tanzania. *Afr Health Sci.* 2011 Mar;11(1):48–57. 21572857 PMC3092321

[42] Mncube BL Mkhize SW : Cervical cancer screening management practices and prevention in uMsunduzi Local Municipality primary care clinics. *Health SA Gesondheid.* 2022;27. 10.4102/hsag.v27i0.1934 36483502 PMC9724042

[43] Denny L Anorlu R : Cervical cancer in Africa. *Cancer Epidemiol Biomarkers Prev.* 2012 Sep;21(9):1434–1438. 10.1158/1055-9965.EPI-12-0334 22806169

[44] Denny L Kuhn L Hu CC : Human papillomavirus-based cervical cancer prevention: long-term results of a randomized screening trial. *J Natl Cancer Inst.* 2010 Oct 20;102(20):1557–1567. 10.1093/jnci/djq342 20884893

[45] Getahun F Mazengia F Abuhay M : Comprehensive knowledge about cervical cancer is low among women in Northwest Ethiopia. *BMC Cancer.* 2013 Jan 2;13. 10.1186/1471-2407-13-2 23282173 PMC3559275

[46] Topuridze M Kareli A Javashvili G : Primary healthcare providers’ knowledge, attitudes, and practices regarding cancer screening recommendation and referral in Georgia, 2023. *European Journal of General Practice.* 2025;31(1). 10.1080/13814788.2025.2582292 41284371 PMC12646092

[47] Musa J Achenbach CJ O’Dwyer LC : Effect of cervical cancer education and provider recommendation for screening on screening rates: A systematic review and meta-analysis. *PLoS ONE.* 2017;12. Public Library of Science. 10.1371/journal.pone.0183924 28873092 PMC5584806

[48] Okolie EA Barker D Nnyanzi LA : Factors influencing cervical cancer screening practice among female health workers in Nigeria: A systematic review. *Cancer Rep (Hoboken).* 2022 May;5(5):e1514. 10.1002/cnr2.1514 34313402 PMC9124499

[49] Rosser JI Hamisi S Njoroge B : Barriers to Cervical Cancer Screening in Rural Kenya: Perspectives from a Provider Survey. *J Community Health.* 2015 Aug;40(4):756–761. 10.1007/s10900-015-9996-1 25677728 PMC8162879

[50] Umuago IJ Obiebi IP Eze GU : Improving primary health care workers’ knowledge of cervical cancer and visual inspection screening techniques through competency-based training: prospects for expanding coverage in developing countries. *Int J Community Med Public Health.* 2020 Apr 24;7(5):1637. 10.18203/2394-6040.ijcmph20201960

[51] Kress CM Sharling L Owen-Smith AA : Knowledge, attitudes, and practices regarding cervical cancer and screening among Ethiopian health care workers. *Int J Womens Health.* 2015;7:765–772. 10.2147/IJWH.S85138 26261427 PMC4527576

[52] Chitha W Sibulawa S Funani I : A cross-sectional study of knowledge, attitudes, barriers and practices of cervical cancer screening among nurses in selected hospitals in the Eastern Cape Province, South Africa. *BMC Womens Health.* 2023 Mar 9;23(1):94. 10.1186/s12905-023-02251-0 36894910 PMC9996860

[53] Allanson ER Schmeler KM : Cervical Cancer Prevention in Low- and Middle-Income Countries. *Clin Obstet Gynecol.* 2021 Sep 1;64(3):501–518. 10.1097/GRF.0000000000000629 34120126 PMC8324570

[54] Sankaranarayanan R Ramadas K Qiao YL : Early Detection of Cancer in Primary Care in Less-Developed Countries. *Cancer Control.* 2013;68–72. Reference Source

[55] Awolude OA Oyerinde SO Akinyemi JO : Screen and triage by community extension workers to facilitate screen and treat: task-sharing strategy to achieve universal coverage for cervical cancer screening in nigeria. *J Glob Oncol.* 2018 Jul;4:1–10. 10.1200/JGO.18.00023 30085882 PMC6223525

[56] Shastri SS Mittra I Mishra GA : Effect of VIA screening by primary health workers: randomized controlled study in Mumbai, India. *J Natl Cancer Inst.* 2014 Mar;106(3):dju009. 10.1093/jnci/dju009 24563518 PMC3982783

[57] World Health Organization: *Comprehensive Cervical Cancer Control: A guide to essential practice.* Geneva: Second Edition 2014. 25642554

[58] Sigfrid L Murphy G Haldane V : Integrating cervical cancer with HIV healthcare services: A systematic review. *PLoS One.* 2017;12(7):e0181156. 10.1371/journal.pone.0181156 28732037 PMC5521786

[59] Altunkurek ŞZ Mohamed SH Şahin E : Knowledge and attitudes of healthcare professionals working in a training and research hospital on early diagnosis of cervical cancer (a Somalia example): cross-sectional study. *BMC Women’s Health.* 2022 Dec 1;22(1). 10.1186/s12905-022-01808-9 35698067 PMC9195217

[60] Adejimi A : Gaps in Knowledge and Practice of Cervical Cancer Screening among Primary Health Care Workers in Lagos, Nigeria. *F1000 Zenodo.* 2026 Apr.

