# Supplementary material for: Gaps in Knowledge and Practice of Cervical Cancer Screening among Primary Health Care Workers in Lagos State, Nigeria: A Cross-Sectional Study
Source: F1000Res. 2026 Apr 28;15:629. [Version 1] doi: 10.12688/f1000research.180107.1 (PMC13563006; doi:10.12688/f1000research.180107.1)
Supplement: Peer review history

# Reviewer report: Reviewer response for version 1

**DOI:** 10.5256/f1000research.198691.r510354

**Published:** 2026-09-01

**Authors:** Chizoma Millicent Ndikom

**Affiliations:** 1 University of Ibadan, Ibadan, Oyo, Nigeria

**Comments**

- Gaps in Knowledge and Practice of Cervical Cancer Screening among Primary Health Care Workers in Lagos State, Nigeria: A Cross-Sectional Study
- This is very interesting, relevant, written, timely and well-structured paper.
- This study assessed knowledge, attitudes, and practices concerning cervical cancer screening among PHC workers in Lagos State.
- The study utilised a descriptive cross-sectional study was quite appropriate for the study. The population of 209 PHC workers selected using multistage sampling showed a good coverage. The use structured questionnaire for Data collection is appropriate for a cross-sectional sturdy.
- Also the statistical test for Data analyses using descriptive statistics and chi-square tests is suitable,
- The results were well presented using appropriate tables according to the objectives and variables in the study.
- The study provided information on the status of knowledge and practice of health workers in the areas of cervical cancer screening.  The study identified the need to strengthen  healthcare workers’ capacity, improve infrastructure as well as  integrate cervical cancer  screening into routine Primary Health Care  services  in order to enhance uptake.   

**Minor corrections**

 Globally, it ranks among the most common cancers affecting women, and it continues as a leading cause of cancer-related deaths in low- and middle-income countries. ^1^

  The HPV is a common sexually transmitted infection that affects a large proportion of sexually active individuals worldwide.  

*(Add full stop to the first sentence. Generally minimize starting sentences with abbreviation to start sentences).*

 These is a very beautiful paper, and I hereby propose that it be accepted with minor corrections

Is the work clearly and accurately presented and does it cite the current literature?

Yes

If applicable, is the statistical analysis and its interpretation appropriate?

Yes

Are all the source data underlying the results available to ensure full reproducibility?

Yes

Is the study design appropriate and is the work technically sound?

Yes

Are the conclusions drawn adequately supported by the results?

Yes

Are sufficient details of methods and analysis provided to allow replication by others?

Yes

Reviewer Expertise:

Women's health with focus on Cervical cervical prevention and early detection.

I confirm that I have read this submission and believe that I have an appropriate level of expertise to confirm that it is of an acceptable scientific standard.

# Reviewer report: Reviewer response for version 1

**DOI:** 10.5256/f1000research.198691.r484904

**Published:** 2026-08-27

**Authors:** Esther Awazzi Envuladu

**Affiliations:** 1 University of Jos, Jos, Plateau, Nigeria

The topic is relevant and it is a subject of public health concern, not only in Nigeria but globally.

 The manuscript is clear and well written. The different sections are also well presented. However, there is a need for some editing. For instance, one of the sentences began with " Because", which is not appropriate.

 It is not clear if the "other PHC staff" included in the study whose designations were not mentioned, actually qualify to be part of the study. Who are they? do they consult or see patients? 

 The results on the availability of Cervical cancer screening services should not be part of the report on the knowledge of cervical cancer screening as reported in the manuscript.

 for the practices, I do not agree that all the healthcare workers should be assessed for the screening practices because it is a specialised procedure conducted by trained personnel. people in the laboratory for instance will not be expected to carry out the screening process.

 Overall, it is a good study and recommended for indexing.

Is the work clearly and accurately presented and does it cite the current literature?

Yes

If applicable, is the statistical analysis and its interpretation appropriate?

Yes

Are all the source data underlying the results available to ensure full reproducibility?

Yes

Is the study design appropriate and is the work technically sound?

Yes

Are the conclusions drawn adequately supported by the results?

Yes

Are sufficient details of methods and analysis provided to allow replication by others?

Yes

Reviewer Expertise:

I am a public health physician and sexual and reproductive health expert

I confirm that I have read this submission and believe that I have an appropriate level of expertise to confirm that it is of an acceptable scientific standard.

# Reviewer report: Reviewer response for version 1

**DOI:** 10.5256/f1000research.198691.r490789

**Published:** 2026-06-23

**Authors:** Zheng Hu

**Affiliations:** 1 Wuhan University, Wuhan, China

Reviewer Comments

 It was a pleasure to review the interesting article by Adebola Afolake Adejimi et al. This study investigated the knowledge, attitudes, and practices (KAP) regarding cervical cancer screening among primary healthcare (PHC) workers in Lagos State, Nigeria. The topic is relevant to the WHO Cervical Cancer Elimination Strategy and addresses an important public health issue in low- and middle-income countries (LMICs). However, the study is a conventional cross-sectional KAP survey and presents several methodological and analytical limitations related to study design, questionnaire development, statistical analysis, and interpretation of findings. These issues need to be further improved.

 Major:

 1. Materials and methods: Study Design and population

 This study employed a multistage sampling approach in which three LGAs were selected from the 20 LGAs in Lagos State, followed by the selection of three PHCs from each LGA, and subsequently all eligible PHC workers were selected.

 Although simple random sampling was reportedly used, important differences among PHCs were not considered. For example, some PHCs may routinely provide cervical cancer screening services, whereas others may not. This factor may influence healthcare workers’ exposure to cervical cancer screening activities and consequently affect their knowledge and practice levels.

 Furthermore, given the multistage sampling design, respondents within the same PHC may share similar training experiences and working environments. The authors should discuss the potential clustering effect and its possible influence on variance estimation.

 2. Materials and methods: Sample Size Determination

 The rationale for the sample size calculation requires further clarification. The study population included medical doctors, nurses, Community Health Extension Workers (CHEWs), laboratory scientists, and other PHC personnel. However, the sample size calculation was based on an estimated proportion of cervical cancer screening knowledge (89.7%) derived from a previous study conducted among female healthcare workers in Plateau State.

 Because the reference population differs substantially from the current study population, the appropriateness of using this estimate should be justified.

 3. Materials and methods: Data analysis 

 The use of a 50% cutoff to classify respondents as having “good knowledge,” “positive attitude,” and “good practice” lacks methodological justification. The authors should provide supporting references or explain the rationale for selecting this threshold.

 Moreover, in addition to categorical classification, reporting continuous KAP scores (mean ± SD) may improve interpretability.

 4. Results: Knowledge of cervical cancer screening

 The presentation of Tables 1 and 2 is unnecessarily detailed. The authors may consider summarizing key knowledge indicators (e.g., the number and percentage of correct responses) and moving detailed questionnaire items to supplementary materials.

 In Table 1, it is unclear whether the reported lack of cervical cancer screening services reflects actual unavailability within the selected PHCs or merely a lack of awareness among respondents. This distinction should be clarified.

 The rationale for including knowledge of treatment (Table 3) in a screening-focused KAP survey should be explained. Since treatment-related knowledge is likely to vary substantially among professional groups, stratified analyses by professional category should be considered.

 5. Results: Practice of cervical cancer screening

 The definition of practice may introduce professional-role bias. The authors primarily evaluated practice based on whether respondents had ever performed cervical cancer screening. However, screening procedures are not routine responsibilities for all professional groups included in the study, particularly laboratory scientists. Therefore, lower practice scores may partly reflect differences in professional roles rather than deficiencies in competence or awareness. Given the substantial heterogeneity among professional categories, stratified analyses should be considered.

 6. Results: Factors associated with knowledge, attitude, and practice of cervical cancer screening

 The use of Chi-square tests does not identify independent predictors of KAP outcomes. Several variables reported as significant, including professional category, duration of practice, and income level, are likely interrelated.

 Multivariable logistic regression models are recommended to identify independent predictors of knowledge, attitudes, and practices after adjustment for potential confounding variables.

 7. Discussion:

 The discussion remains largely descriptive and primarily focuses on comparing the findings with previous studies.

 One of the most important findings of this study is the substantial discrepancy between knowledge/attitudes and actual screening practice. However, the manuscript does not adequately explore the relationships among these three domains. Analyses examining whether knowledge predicts practice, or whether attitudes mediate the relationship between knowledge and practice, would substantially strengthen the manuscript.

 Furthermore, the discussion provides only limited exploration of the underlying reasons for the observed implementation gap. The recommendations mainly focus on training, guideline dissemination, task shifting, equipment provision, and referral systems. While these recommendations are reasonable, they are relatively conventional. The authors may consider discussing additional WHO-endorsed strategies, such as HPV self-sampling and other emerging screening approaches that may be particularly relevant in under-served LMIC settings.

 Minor:

 1. A dedicated table summarizing the socio-demographic characteristics of participants would improve clarity and facilitate interpretation of the study population.

 2. The manuscript should clearly indicate whether missing data were present and describe how incomplete questionnaires were handled during data analysis.

 3. The inclusion of multiple professional categories may be considered a strength of the study. However, this advantage is not fully utilized because most analyses were not stratified by profession. Additional subgroup analyses could provide more meaningful insights.

Is the work clearly and accurately presented and does it cite the current literature?

Yes

If applicable, is the statistical analysis and its interpretation appropriate?

Partly

Are all the source data underlying the results available to ensure full reproducibility?

Yes

Is the study design appropriate and is the work technically sound?

Yes

Are the conclusions drawn adequately supported by the results?

Partly

Are sufficient details of methods and analysis provided to allow replication by others?

Partly

Reviewer Expertise:

cervical cancer screening

I confirm that I have read this submission and believe that I have an appropriate level of expertise to confirm that it is of an acceptable scientific standard, however I have significant reservations, as outlined above.
